# Foot and Ankle Biomechanics in Individuals With Patellofemoral Pain: A Systematic Review and Meta‐Analysis

**DOI:** 10.1155/tsm2/7859730

**Published:** 2026-07-20

**Authors:** Luiza Pizarro Chaffe, José Luis Flor Silva, Nicolas da Silva Pereira, Klauber Dalcero Pompeo, Emmanuel Souza da Rocha, Rodrigo Rodrigues

**Affiliations:** ^1^ Graduate Program in Health Science, Federal University of Rio Grande, Rio Grande, State of Rio Grande do Sul, Brazil, furg.br; ^2^ Instituto Federal de Educação, Ciência e Tecnologia Sul-rio-grandense - Campus Sapiranga, Sapiranga, State of Rio Grande do Sul, Brazil; ^3^ Graduate Program in Health Technology, Pontifícia Universidade Católica do Paraná, Curitiba, State of Paraná, Brazil, pucpr.br; ^4^ Exercise Research Laboratory, Federal University of Rio Grande do Sul, Porto Alegre, State of Rio Grande do Sul, Brazil, ufrgs.br

**Keywords:** distal joints, kinematics, plantar pressure, range of motion, rearfoot

## Abstract

Patellofemoral pain (PFP) is a common, multifactorial musculoskeletal condition that primarily affects young and physically active adults. Although previous studies have emphasized proximal and local impairments, the contribution of distal joint biomechanics remains unclear. This systematic review and meta‐analysis aimed to determine whether biomechanical and clinical parameters of the foot and ankle differ between individuals with and without PFP. A systematic search was conducted in PubMed, ScienceDirect, SciELO, and SPORTDiscus. Studies comparing adults with and without PFP in distal joint outcomes, including muscle strength, kinematics, range of motion (ROM), plantar pressure, and clinical measures (e.g., navicular drop and foot mobility), were included. Methodological quality was assessed using a modified Downs and Black scale. Random‐effects meta‐analyses were performed using Hedges’ g, and the level of evidence for each outcome was classified according to predefined criteria based on methodological quality, statistical significance, and between‐study heterogeneity. Forty‐two studies were included in the systematic review, and 38 were included in the meta‐analysis, comprising 2314 participants (PFP: *n* = 1211; controls: *n* = 1103). Individuals with PFP demonstrated greater navicular drop (SMD = −0.46; *p* = 0.004), supported by strong evidence, although this finding was based on only four studies (PFP: *n* = 84; controls: *n* = 84). Greater rearfoot eversion during landing and step‐down tasks, as well as greater ankle dorsiflexion during the step‐down task, were also observed; however, these findings were supported by very limited evidence. No significant between‐group differences were identified for ankle dorsiflexion ROM measured in clinical tests, foot posture index scores, or plantar pressure distribution. Overall, altered distal biomechanics, particularly greater navicular drop, appear to be associated with PFP. However, the available evidence remains limited for most outcomes, and the predominantly case–control design of the included studies precludes causal inference.

## 1. Introduction

Patellofemoral pain (PFP) is characterized by pain around or behind the patella during activities such as jumping, squatting, running, and stair climbing [1] and has a prevalence of approximately 25% among physically active young adults [2]. Although PFP is multifactorial, alterations in lower‐limb alignment during motor tasks, including the presence of dynamic valgus, have been associated with pain [3, 4]. Dynamic knee valgus is defined as increased ipsilateral trunk lean, hip adduction and internal rotation, and rearfoot eversion [3], resulting in medial displacement of the knee during closed‐kinetic‐chain movements [5]. Cross‐sectional studies have demonstrated associations between proximal and distal mechanical parameters and poor lower‐limb alignment during dynamic activities [6–9], which may increase patellofemoral joint stress and pain [3].

Systematic reviews with meta‐analysis have shown differences between individuals with and without PFP in strength, kinematics, muscle activation, and muscle morphology. Individuals with PFP show lower hip muscle strength [10, 11], greater trunk flexion and ipsilateral trunk lean during landing and single‐leg squat tasks [12], greater peak hip adduction during running and single‐leg squat activities [11], lower gluteus medius activation [13], delayed electromyographic onset of the vastus medialis and higher activation of the biceps femoris [14], and differences in muscle morphology at hip, knee, and ankle/foot joints [15, 16].

Given that most pain‐provoking tasks in individuals with PFP involve weight‐bearing, the ankle and foot joints contribute to the movement chain. The ankle and foot joints absorb and distribute loads and influence stability, locomotion efficiency, and the development of musculoskeletal dysfunctions [17]. Cross‐sectional studies have shown that individuals with PFP present greater foot mobility [18], increased rearfoot eversion [19], higher plantar pressure [20], and greater navicular drop [18] compared with individuals without PFP. Such alterations can lead to biomechanical compensations and changes in lower‐limb alignment that affect the knee joint and are associated with PFP [3]. However, only two systematic reviews have focused on the ankle and foot joints, reporting limited evidence regarding ankle–foot alignment in individuals with PFP [21], increased rearfoot eversion in females during running or walking [22], and differences in distal muscle activation and morphology between individuals with and without PFP [13]. Importantly, these reviews did not provide a comprehensive quantitative synthesis across multiple biomechanical and clinical outcomes of ankle and foot joints in individuals with PFP.

Given the scarcity of evidence that exercise programs targeting ankle and foot muscles combined with knee exercise are as effective as traditional programs focused on trunk and hip muscles combined with knee exercises or on knee muscles alone for reducing pain and improving function in individuals with PFP [23, 24], understanding ankle and foot functional differences between individuals with and without PFP may support the development of more precise rehabilitation strategies. Therefore, this study is novel in that it systematically and quantitatively evaluates a broad range of biomechanical and clinical parameters of the ankle–foot complex in individuals with and without PFP, while also exploring potential sources of heterogeneity. This information may contribute to a better understanding of distal factors associated with PFP and may help inform future research and clinical decision‐making.

## 2. Methods

This systematic review and meta‐analysis was registered in the International Prospective Register of Systematic Reviews (PROSPERO; CRD42023443541) and conducted in accordance with the Preferred Reporting Items for Systematic Reviews and Meta‐Analyses (PRISMA) statement [25].

### 2.1. Eligibility Criteria

Case–control studies, prospective studies, and clinical trials comparing distal joint outcomes between individuals with PFP and those without PFP (control group, CG) were included. The inclusion criteria were (i) studies involving human participants; (ii) participants diagnosed exclusively with PFP; (iii) assessment of biomechanical or functional parameters of the ankle and foot joints, including strength, kinematics, range of motion (ROM), and clinical tests such as the navicular drop and foot mobility; and (iv) publication in English, Spanish, or Portuguese.

Studies were excluded if they were reviews, conference proceedings, letters, or symposium abstracts, as well as research involving participants with other musculoskeletal conditions, studies without distal joint measurements, or studies lacking a healthy control group.

### 2.2. Search Strategy

The search was conducted in the PubMed, SciELO, ScienceDirect, and SPORTDiscus databases, with no date restrictions. Reference lists of the included studies were also analyzed to identify additional relevant research. Studies published up to June 2026 were considered. An updated search for gray literature was also performed in university digital repositories and institutional libraries to identify potentially eligible theses, dissertations, and other unpublished academic materials. MeSH terms and keywords related to PFP and its biomechanical and functional outcomes, such as kinematics, strength, ROM, mobility, and plantar pressure, were utilized. In SciELO, ScienceDirect, SPORTDiscus, and institutional libraries, simplified searches were performed by combining main topic keywords (e.g., “Patellofemoral Pain Syndrome” and “Range of Motion”). Additionally, a manual search was conducted in the reference lists of studies related to PFP. Boolean operators “AND” and “OR” were applied to combine the terms. A full description of the search strategy used in Medline via PubMed is presented in Table 1.

**TABLE 1:** Full description of the search strategy to be used in Medline via the PubMed database.

TABLES(“Patellofemoral Pain Syndrome”[Mesh] OR “Pain Syndrome, Patellofemoral” OR “Anterior Knee Pain Syndrome” OR “Patellofemoral Syndrome” OR “Patellofemoral Pain” OR “Pain, Patellofemoral” OR “Patellofemoral Pains”) AND (“Electromyography”[Mesh] OR “Electromyographies” OR “Surface Electromyography” OR “Electromyographies, Surface” OR “Electromyography, Surface” OR “Surface Electromyographies” OR “Electromyogram” OR “Electromyograms” OR “neural drive” OR “muscle coordination” OR “muscle architecture” OR “muscle thickness” OR “muscle activation” OR “Muscle Strength”[Mesh] OR “Strength, Muscle” OR “Dynamometer, Muscle Strength” OR “Dynamometers, Muscle Strength” OR “Muscle Strength Dynamometers” OR “muscle volume” OR “Muscular Atrophy”[Mesh] OR “Atrophies, Muscular” OR “Atrophy, Muscular” OR “Muscular Atrophies” OR “Atrophy, Muscle” OR “Atrophies, Muscle” OR “Muscle Atrophies” OR “Muscle Atrophy” OR “Neurogenic Muscular Atrophy” OR “Atrophies, Neurogenic Muscular” OR “Atrophy, Neurogenic Muscular” OR “Muscular Atrophies, Neurogenic” OR “Muscular Atrophy, Neurogenic” OR “Neurogenic Muscular Atrophies” OR “Neurotrophic Muscular Atrophy” OR “Atrophies, Neurotrophic Muscular” OR “Atrophy, Neurotrophic Muscular” OR “Muscular Atrophies, Neurotrophic” OR “Muscular Atrophy, Neurotrophic” OR “Neurotrophic Muscular Atrophies” OR “Range of Motion” OR “ROM” OR “Range of Motion, Articular”[Mesh] OR “Joint Range of Motion” OR “Joint Flexibility” OR “Flexibility, Joint” OR “Passive Range of Motion” OR “Arch Height Index” OR “Navicular Drop” OR “rearfoot eversion” OR “Foot Joints”[Mesh] OR “Foot Joint” OR “Joint, Foot” OR “Joints, Foot” OR “Articulationes pedis” OR “Intermetatarsal Joint” OR “Intermetatarsal Joints” OR “Joint, Intermetatarsal” OR “Joints, Intermetatarsal” OR “Foot”[Mesh] OR “Feet” OR “Ankle Joint”[Mesh] OR “pronation” OR “plantar pressure” OR “Midfoot Height Mobility”)

### 2.3. Study Selection

The results from each database were exported to EndNote for duplicate removal. Two independent authors screened the titles and abstracts (L.P.C. and J.L.F.S.), excluding studies that did not meet the eligibility criteria. In cases of disagreement, a third reviewer was consulted (R.R.). The selected articles then had their full texts assessed to determine final inclusion.

### 2.4. Data Extraction

Data were extracted using a form created by the researchers, including publication information (author and year), demographic data (sex, age, body mass, height, and physical activity profile), and baseline symptoms (symptom duration, usual pain intensity, unilateral/bilateral symptoms, pain intensity during activities, and physical function assessed by questionnaire). Means and standard deviations for both groups (with and without PFP) were recorded for all available outcomes of interest. If a study included more than two groups (e.g., another musculoskeletal condition or another intervention), only data from the relevant groups were extracted. In prospective studies or clinical trials, only data from the group that had already developed PFP and the group without PFP, as well as pre‐intervention assessments, were extracted.

Data were also extracted for the meta‐analysis. When data were presented only in figures and not in text or tables, the study authors were contacted. If no response was received within 1 month, those values were extracted using dedicated image analysis software (ImageJ, National Institutes of Health, Bethesda, Maryland) to obtain mean and standard deviation values [26]. When studies presented multiple tasks or different phases of the same task, all values were extracted. In addition, whenever possible, results for men and women were reported separately.

### 2.5. Methodological Quality Assessment

Methodological quality was assessed using a modified version of the Downs and Black checklist [27] as previously applied in similar studies [13, 28, 29]. The modified checklist comprised 15 items, excluding questions related to randomization and interventions from the original instrument. The maximum possible score was 16 points, and studies were classified as having low (< 9 points), moderate (10–11 points), or high (≥ 12 points) methodological quality [13, 28, 29].

### 2.6. Data Analysis

Data were pooled for meta‐analysis when at least two studies assessed the same outcome. For muscle strength, ROM, and kinematic variables, separate analyses were performed according to the specific movement evaluated (e.g., inversion, eversion, and dorsiflexion). Kinematic outcomes were further stratified by task (e.g., walking, running, and step‐down). When studies reported outcomes separately for different phases of the same movement (e.g., throughout the gait cycle) or for distinct subgroups within the same population (e.g., participants with high and low pain levels or longer and shorter symptom duration), data were combined. Combined means were calculated as weighted averages according to subgroup sample sizes, and combined standard deviations were calculated using recommended methods that account for within‐group variability and differences between subgroup means. The resulting pooled estimates were then included in the meta‐analysis.

In the meta‐analysis, a random‐effects model was used. Standardized mean differences (SMD), calculated using Hedges’ g, were reported along with 95% confidence intervals in forest plots, with *p* < 0.05 considered statistically significant [30]. SMDs were categorized as having small (up to 0.59), medium (0.60–1.19), or large (≥ 1.20) effect sizes. Statistical heterogeneity was assessed using the inconsistency test (*I* ^2^), with values above 25% and 50% considered indicative of moderate and high heterogeneity, respectively [31]. To explore between‐study heterogeneity, subgroup analyses were performed according to sex or assessment method (e.g., passive vs. weight‐bearing ROM tests) whenever sufficient data were available. In addition, leave‐one‐out sensitivity analyses were conducted by sequentially removing one study at a time to evaluate the influence of individual studies on the pooled effect estimates. All statistical analyses were performed using Review Manager software (RevMan, Version 5.3).

The level of evidence was determined according to criteria adapted from previous meta‐analyses [14, 22] and classified as follows: (i) strong evidence, defined as a statistically significant and homogeneous pooled effect (*I* ^2^ ≤ 50%) from at least three studies, including at least two high‐quality studies; (ii) moderate evidence, defined as either a statistically significant but heterogeneous pooled effect (*I* ^2^ > 50%) from multiple studies including at least one high‐quality study or a statistically significant and homogeneous pooled effect (*I* ^2^ ≤ 50%) from multiple moderate‐ or low‐quality studies; (iii) limited evidence, defined as findings from one high‐quality study or from multiple moderate‐ or low‐quality studies with statistically significant but heterogeneous results (*I* ^2^ > 50%); (iv) very limited evidence, defined as findings from a single moderate‐ or low‐quality study; and (v) conflicting evidence, defined as nonsignificant and heterogeneous findings (*I* ^2^ > 50%) pooled from multiple studies, regardless of methodological quality [32].

## 3. Results

### 3.1. Included Studies

**FIGURE 1:**
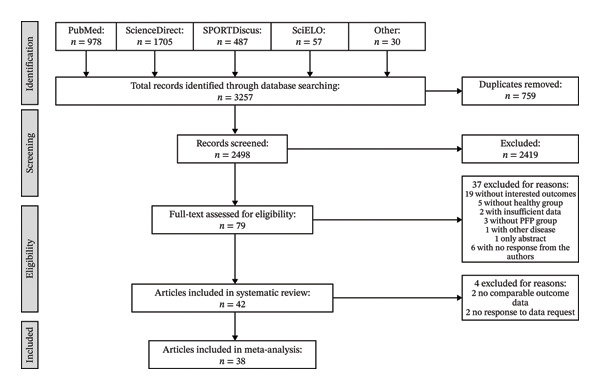
Flow chart of included studies according to PRISMA.

The initial search returned 3257 studies from the databases. After removing duplicates, 2498 titles and abstracts were screened. Of these, 79 full‐text articles were assessed for eligibility. After applying the inclusion and exclusion criteria, 42 studies were included in the systematic review. Among the included studies, 41 used a case–control design, while only one adopted a prospective design [33]. For the meta‐analysis, 2 studies could not be included because they could not be compared with at least one other study assessing the same outcome [34, 35], and two studies were excluded because the authors did not respond to the data request [33, 36]. Thus, 38 studies were included in the meta‐analysis (Figure 1). Twenty‐two studies measured rearfoot eversion [19, 37–57]. Ten studies measured dorsiflexion ROM using kinematics assessment during weight‐bearing tasks [40, 45, 54, 55, 57–62]. Seven studies assessed ankle dorsiflexion ROM using goniometry or functional tests [18, 44, 63–67]. Five studies measured plantar pressure [20, 42, 64, 68, 69]. Four studies assessed navicular drop [18, 53, 56, 70], and four studies assessed foot posture index [18, 63, 66, 71]. We cannot perform meta‐analyses involving muscle strength.

### 3.2. Characteristics of the Included Studies

#### 3.2.1. Participants

A total of 2314 participants were included (PFP, *n* = 1211; CG, *n* = 1103). Sample sizes ranged from 18 to 66 participants per group, with a predominance of females (70%) and ages between 18 and 35 years. Body mass index (BMI) was mostly between 19 and 27 kg/m^2^. Symptom duration varied widely, ranging from 1 month to 30 years; however, most studies reported symptoms persisting for more than one year. Bilateral symptoms were identified in only three studies.

Regarding physical activity level, approximately 50% of the studies reported this information, and participants were mostly classified as recreational or moderately active. Eleven studies evaluated runners with different weekly training volumes. Pain intensity varied widely, ranging from 0.8 to 6 cm on a Visual Analog Scale. Functional capacity was assessed in more than half of the studies, most commonly using the anterior knee pain scale, with scores ranging from 49.6 to 85 (on a scale where the score ranges from 0 to 100, where 100 represents the absence of pain) (Table 2).

**TABLE 2:** Characteristics of the included studies.

Study	M	F	Group	Age (y)	Height (cm)	Body mass (kg)	Physical activity	Pain	Duration of symptoms	Functional status	Bilateral symptoms
Aliberti et al. [20]	4	26	PFP	30 ± 7	165 ± 9	63 ± 11	3 times/wk	1.6 ± 2.3	4 ± 3 y	70 (median)^§^	NI
	5	39	CG	30 ± 8	165 ± 8	60 ± 11	3 times/wk	0	—	98 (median)^§^	NI

Aliberti et al. [69]	2	20	PFP	30 ± 7	165 ± 9	63 ± 12	2 ± 1 times/wk	1.7 ± 2.3	4 ± 3 y	70 (median)^§^	NI
	3	32	CG	29 ± 7	164 ± 8	60 ± 11	3 ± 1 times/wk	0	—	98 (median)^§^	NI

Baellow et al. [66]	9	26	PFP	20.5 ± 3.8	170.8 ± 11.9	73.3 ± 26.6	—	2 ± 1.6	51.1 ± 39.7 m	77.3 ± 9.8^∗∗∗^	NI
	9	26	CG	20.4 ± 3.2	169.6 ± 9.1	64.8 ± 11.5	—	0	—	100^∗∗∗^	NI

Barton et al. [18]	5	15	PFP	22.8 ± 4.1	167.9 ± 6.8	66.8 ± 11.3	3682 ± 1642 mets^#^	≥ 3	At least 6 weeks	—	—
	5	15	CG	21.9 ± 3.5	169.9 ± 8.3	63.9 ± 14.0	3028 ± 1400 mets^#^	0	—	—	—

Barton et al. [37]	5	21	PFP	25.1 ± 4.6	168.6 ± 8.4	66.7 ± 12.8	5801 ± 2991 mets^#^	≥ 3	At least 6 weeks	—	—
	4	16	CG	23.4 ± 2.3	171.1 ± 8.4	66.0 ± 15.4	4761 ± 3937 mets^#^	0	—	—	—

Bramah et al. [54]	28	44	PFP	34.5 ± 9.4	173.5 ± 8.5	64.4 ± 9.6	18.6 ± 6.9 mi/wk	> 3	NI	—	—
	15	21	CG	33.2 ± 8.4	171.6 ± 7.3	60.8 ± 8.4	60.5 ± 23.2 mi/wk	0	—	—	—

Callaghan et al. [38]	0	15	PFP	27 ± 6.5	—	61.5 ± 17.5	—	—	1.7 ± 1.4 years	—	0%
	0	15	CG	23.5 ± 3.2	—	59.7 ± 6.9	—	—	—	—	—

Carvalho‐Silva. [56]	0	20	PFP	22.8 ± 2.8	162 ± 7	56.8 ± 10.0	—	6 ± 1.8	28 ± 18 m	78.9 ± 17.2^∗∗∗^	65%
	0	20	CG	24.1 ± 2.6	163 ± 6	61.9 ± 10.0	—	0	0	98.4 ± 2.3^∗∗∗^	—

Dag et al. [64]	6	9	PFP	21.3 ± 1.8	167.8 ± 7.2	60.1 ± 8.6	7612 ± 5231 mets^#^	5.7 ± 1.6	18.3 ± 15.9 m	—	0%
	6	9	CG	21.4 ± 2.1	170.8 ± 8.7	63.5 ± 9.4	8952 ± 7427 mets^#^	0	—	—	—

Dierks et al. [39]	5	15	PFP	28.1 ± 5.9	170.5 ± 6.8	62.1 ± 8.9	Runners 27.3 km/wk	≥ 3	At least 2 m	—	—
	5	15	CG	26.5 ± 6.4	168.7 ± 6.3	60.3 ± 7.8	Runners 24.6 km/wk	0	—	—	—

Dingenen et al. [59]	5	13	PFP	31.3 ± 6.8	170.5 ± 8.4	66.1 ± 10.5	11.6 ± 12.4 km/wk	—	14.3 ± 19.3 m	72.3 ± 5.6^∗∗^	—
	7	17	CG	35.8 ± 16.4	169.5 ± 8.4	66 ± 11	37.4 ± 16.6 km/wk	—	—	80^∗∗^	—

Duffey et al. [48]	68	31	PFP	23.2 ± 4.5	168.3 ± 6.1	68.5 ± 9.2	15–20 km/wk	5.5 ± 1.3	—	—	—
	53	17	CG	22.5 ± 3.9	170.2 ± 6.2	66.7 ± 8.4	20–25 km/wk	0	—	—	—

Fu et al. [62]	14	9	PFP	23.8 ± 3.5	172 ± 0.08	64.1 ± 8.4	> 10 km/wk	85 ± 6.8^∗∗∗^	At least 3 m	—	—
	9	10	CG	23.3 ± 2.3	171 ± 0.1	63.5 ± 8.9	> 10 km/wk	0	—	—	—

Gomes et al. [36]	0	18	PFP	21.06 ± 1.63	161 ± 0.05	58.91 ± 8.93	Physically active^#^	5.1 ± 1.3	At least 3 m	—	—
	0	18	CG	22.89 ± 4.04	163 ± 0.09	58.42 ± 9.97	Physically active^#^	0	—	—	—

Hassan et al. [65]	27	43	PFP	25.6 ± 3.7	165 ± 0.05	62.2 ± 6.4	4–5 times/wk	5.1 ± 1.3	—	—	—
	35	35	CG	26.1 ± 4	167 ± 0.06	63 ± 6.2	4–5 times/wk	0	—	—	—

Jaffri et al. [71]	11	19	PFP	20.2 ± 3.3	161 ± 11.7	74.7 ± 27.6	—	4.7 ± 1.4	46.7 ± 28.3 m	77.1 ± 9.34	—
	11	19	CG	20.3 ± 3.3	169 ± 9.3	64 ± 11	—	0	0	100	—

Kedroff et al. [70]	6	5	PFP	23.7 ± 2.8	170 ± 10	69.6 ± 11.2	—	2 (1–3)	58.6 ± 71.9	80 (75–83)	81.8%
	6	5	CG	25.0 ± 4.6	170 ± 10	67.6 ± 14.7	—	0	—	100	—

Laprade [53]	22	11	PFP	30.8 ± 8.5	174 ± 8.8	77 ± 14.5	—	—	—	—	—
	22	11	CG	31 ± 8.4	177 ± 6.5	75 ± 10.5	—	—	—	—	—

Levinger et al. [49]	0	15	PFP	33.9 ± 10.2	167 ± 0.6	69.7 ± 19.8	—	—	7.96 (1–30) y	—	—
	0	15	CG	27.1 ± 10.8	166 ± 0.7	62.5 ± 6.4	—	—	—	—	—

Levinger et al. [40]	0	15	PFP	38.4 ± 10.1	166 ± 6	70.6 ± 18.2	3 h/wk	2	11 (1.5–30) y	—	—
	0	15	CG	25.1 ± 8.7	166 ± 6.5	61.3 ± 7.6	4.1 h/wk	0.0	—	—	—

Lopes Ferreira et al. [63]	0	23	PFP	22.9 ± 3	163 ± 0.5	58.8 ± 6.7	Physically active^#^	5.67 ± 1^∗^	19.4–74	64.4–76.3^∗∗∗^	—
	0	28	CG	25.2 ± 3.1	163 ± 0.3	58.2 ± 4.5	Physically active^#^	0	—	100^∗∗∗^	—

Luza et al. [42]	0	23	PFP	23 ± 6	165 ± 7.5	59.8 ± 8.1	—	≥ 2	—	—	—
	0	28	CG	21 ± 4	164 ± 0.5	59.1 ± 8.1	—	0	—	—	—

Luz et al. [41]	16	11	PFP	22 ± 5.7	165 ± 0.7	58.9 ± 8.1	Running (15 km/wk)	≥ 2	> 3 m	78^∗∗∗^	—
	16	11	CG	21.5 ± 3.8	164 ± 0.5	59.5 ± 8.3	Running (15 km/wk)	0	—	100^∗∗∗^	—

Morita & Navega [60]	0	30	PFP	21.3 ± 3.4	162 ± 0.7	60.6 ± 13.4	—	4.8 ± 1.4	> 8 weeks	76.4 ± 7.7^∗∗∗^	—
	0	30	CG	22.9 ± 3.7	162 ± 0.7	62.4 ± 13.3	—	—	—	—	—

Noehren et al. [45]	0	16	PFP	27 ± 6	164 ± 5	57.4 ± 4.6	Running (23 ± 10 km/wk)	≥ 3	> 2 m	—	—
	0	16	CG	25 ± 4	165 ± 7	58.7 ± 6.5	Running (35 ± 16 km/wk)	—	—	—	—

Messier et al. [44]	14	10	PFP	30.5 ± 6.1	173.3 ± 6.9	68.2 ± 8.6	Running (42.5 km/wk)	—	—	—	—
	13	11	CG	31.2 ± 5.9	175 ± 6.7	70.1 ± 9.1	Running (44.3 km/wk)	—	—	—	—

Piazza et al. [43]	0	23	PFP	22.4 ± 5.6	165 ± 7	58.9 ± 8.1	—	≥ 2	< 2 years	—	56.5%
	0	28	CG	21.4 ± 3.7	164 ± 5	59.5 ± 8.2	—	0	—	—	—

Piva et al. [34]	13	17	PFP	25.8 ± 6	169 ± 4	76.9 ± 17.4	50%: jumping, pivoting, cutting, football, soccer	3.9 ± 2.2		64.5 ± 18.4^∗∗∗^	—
	13	17	CG	25.7 ± 5.9	170 ± 10.6	68.8 ± 14.2	13.3%: jumping, pivoting, cutting, football, soccer	0		99.8 ± 0.8^∗∗∗^	—

Powers et al. [51]	0	15	PFP	22.0 ± 3.6	169 ± 6	65 ± 8	—	—	—	—	—
	0	15	CG	23.3 ± 2.5	170 ± 5	64 ± 7	—	—	—	—	—

Powers et al. [46]	0	24	PFP	25.4 ± 7.3	165 ± 10.8	63.6 ± 10.1	—	—	—	—	—
	0	18	CG	27.6 ± 4.8	166 ± 7.6	59.6 ± 7.5	—	—	—	—	—

Rathleff et al. [68]	13	10	PFP	25.8 ± 7.4	174 ± 10.9	71.2 ± 14	343.7 ± 272 mi/wk	—	> 6 weeks	—	—
	10	10	CG	26.6 ± 3.1	176 ± 9.2	72.1 ± 14.1	536 ± 205 mi/wk	—	—	—	—

Reis et al. [61]	0	20	PFP	23.5 ± 2.1	171 ± 1.3	55.3 ± 4.8	Physically active^#^	4.9 ± 1.6	> 3 m	—	—
	0	20	CG	23.1 ± 3.3	165 ± 1.2	55.9 ± 7.1	Physically active^#^	0	—	—	—

Reis et al. [55]	0	34	PFP	26.5 ± 8.2	158 ± 5.8	57.2 ± 6.8	—	5.7 ± 1.6	—	66.7 ± 10^∗∗∗^	—
	0	36	CG	24.6 ± 4	163 ± 6.2	55.6 ± 6.1	—	0	—	100	—

Rodrigues et al. [50]	13	4	PFP	29.8 ± 7.2	163 ± 8	60.2 ± 7.7	Runners 12.9 km/wk	—	—	—	—
	10	9	CG	34.2 ± 10.9	172 ± 9	64.6 ± 11.9	Runners 12.9 km/wk	—	—	—	—

Rodrigues et al. [78]	0	15	PFP	26.33 ± 4.18	163 ± 6	66 ± 13.1	2132 ± 1824 mets^#^	2.2 ± 1.2	—	76.3 ± 10.2^∗∗∗^	—
	0	15	CG	29 ± 5.23	164 ± 6	62.2 ± 7.8	2053 ± 1648 mets^#^	0.1 ± 0.4	—	NI	—

Steinberg et al. [33]	0	10	PFP	—	—	—	Dancers	—	> 46.7 m	—	—
	0	10	CG	—	—	—	Dancers	—	—	—	—

Silva et al. [19]	0	29	PFP	21.8 ± 2.7	165 ± 6	65 ± 9.1	4433 ± 437 mets^#^	5.8 ± 2	> 1 m	—	—
	0	25	CG	22.3 ± 3.5	165 ± 4	63.4 ± 6.3	4030 ± 595 mets^#^	0	—	—	—

Silva et al. [47]	0	29	PFP	24.9 ± 4.4	163 ± 0.05	59.5 ± 8.2	—	≥ 3	—	—	—
	0	25	CG	23.5 ± 3.6	164 ± 0.06	58.1 ± 7.5	—	0	—	—	—

Shi et al. [57]	17	9	PFP	22.3 ± 2.5	174 ± 0.08	64.9 ± 9	—	84.3 ± 6.7^∗∗∗^	> 1 year	—	57.1%
	9	7	CG	21.8 ± 2.1	172 ± 0.09	62.4 ± 9.3	—	0	—	—	—

Van Cant et al. [35]	15	6	PFP	21.1 ± 2.6	162 ± 5.8	55.9 ± 7.4	3.8 ± 0.6^$^	—	> 2 m	58.6 ± 11.1^∗∗^	—
	14	7	CG	20.5 ± 2.8	165 ± 5.8	58.3 ± 7.4	3.8 ± 0.6^$^	—	—	78.7 ± 1.9^∗∗^	—

Valle et al. [52]	5	9	PFP	24 ± 5	170 ± 0.9	67 ± 11	—	4 ± 2^∗^	—	74 ± 9^∗∗∗^	—
	3	11	CG	26 ± 5	168 ± 0.6	65 ± 10	—	—	—	—	—

Willson et al. [6]	0	19	PFP	21.3 ± 2.6	168 ± 6	62.9 ± 7.7	Run > 10 mi/wk	> 3	> 2 m	< 85 pts^∗∗∗^	—
	0	20	CG	21.6 ± 4.5	169 ± 9	62.1 ± 8.9	Run > 10 mi/wk	—	—	—	—

*Note:* y: years; m: months.

^#^IPAQ: International Physical Activity Questionnaire.

^$^Free‐time activity (BAQ: Baecke Activity Questionnaire).

^∗∗^LEFS: Lower Extremity Functional Scale.

^∗^NPRS: Numerical Pain Rating Scale.

^§^Lysholm Functional Knee Scale.

^∗∗∗^Anterior Knee Pain Scale.

### 3.3. Methodological Quality Assessment

Fifteen studies (35.7%) were classified as high methodological quality, 24 (57.1%) as moderate quality, and three (7.1%) as low quality, with a mean Downs and Black score of 11.1 (range: 9–13) (Table 3). The methodological quality assessment demonstrated consistent reporting of study objectives, outcomes, and participant characteristics. Nevertheless, important methodological limitations were identified across the included studies, particularly in external validity (0% of studies met the criterion), assessor blinding (0%), and adjustment for confounding factors (0%). These limitations should be considered when interpreting the pooled findings.

**TABLE 3:** Assessment of methodological quality using the modified version of the Downs and Black scale.

Study	1	2	3	5^∗^	6	7	10	11	12	15	16	18	20	21	25	Total
Aliberti et al. [20]	1	1	1	2	1	1	1	0	0	0	1	1	1	1	0	**12**
Aliberti et al. [69]	1	1	1	2	1	1	1	0	0	0	1	1	1	1	0	**12**
Baellow et al. [66]	1	1	1	2	1	1	1	0	0	0	1	1	1	1	0	**13**
Barton et al. [18]	1	1	1	1	1	1	1	0	0	0	1	1	1	1	0	**12**
Barton et al. [37]	1	1	1	1	1	1	1	0	0	0	0	1	1	1	0	**9**
Bramah et al. [54]	1	1	1	1	1	1	0	0	0	0	1	1	1	1	0	**11**
Callaghan et al. [38]	1	1	1	1	1	1	1	0	0	0	1	1	1	0	0	**10**
Carvalho‐Silva et al. [56]	1	1	1	2	1	1	1	0	0	0	1	1	1	1	0	**12**
Dag et al. [64]	1	1	1	2	1	1	1	0	0	0	1	1	1	1	0	**12**
Dierks et al. [39]	1	1	1	1	1	1	1	0	0	0	1	1	1	1	0	**11**
Dingenen et al. [59]	1	1	1	1	0	1	1	0	0	0	1	1	1	0	0	**9**
Duffey et al. [48]	1	1	1	1	1	1	1	0	0	0	1	1	1	1	0	**11**
Fu et al. [62]	1	1	1	1	1	1	1	0	0	0	1	1	1	1	0	**12**
Gomes et al. [36]	1	1	1	1	1	1	1	0	0	0	1	1	1	1	0	**12**
Hassan et al. [65]	1	1	1	1	0	1	1	0	0	0	1	1	1	1	0	**10**
Jaffri et al. [71]	1	1	1	2	1	1	1	0	0	0	1	1	1	1	0	**12**
Kedroff et al. [70]	1	1	1	2	1	1	1	0	0	0	1	1	1	0	0	**11**
Laprade [53]	1	1	1	1	1	1	1	0	0	0	1	1	1	1	0	**11**
Levinger et al. [49]	1	1	1	1	1	1	1	0	0	0	1	1	1	1	0	**11**
Levinger et al. [40]	1	1	1	1	1	1	1	0	0	0	1	1	1	1	0	**11**
Lopes Ferreira et al. [63]	1	1	1	2	1	1	1	0	0	0	1	1	1	1	0	**12**
Luza et al. [42]	1	1	1	1	1	1	1	0	0	0	1	1	1	1	0	**11**
Luz et al. [41]	1	1	1	1	1	1	1	0	0	0	1	1	1	1	0	**11**
Morita & Navega [60]	1	1	1	1	1	1	1	0	0	0	1	1	1	1	0	**11**
Messier et al. [44]	1	1	1	1	1	1	1	0	0	0	1	1	1	1	0	**11**
Noehren et al. [45]	1	1	1	1	1	1	1	0	0	0	1	1	1	1	0	**11**
Piazza et al. [43]	1	1	1	1	1	1	1	0	0	0	1	1	1	1	0	**11**
Piva et al. [34]	1	1	1	2	1	1	1	0	0	0	1	1	1	1	0	**12**
Powers et al. [51]	1	1	1	1	1	1	1	0	0	0	1	1	1	1	0	**11**
Powers et al. [46]	1	1	1	1	1	1	1	0	0	0	1	1	1	1	0	**11**
Rathleff et al. [68]	1	1	1	2	1	1	1	0	0	0	1	1	1	1	0	**12**
Reis et al. [61]	1	1	1	1	1	1	1	0	0	0	1	1	1	1	0	**11**
Reis et al. [55]	1	1	1	1	1	1	1	0	0	0	1	1	1	1	0	**11**
Rodrigues et al. [78]	1	1	1	2	1	1	1	0	0	0	1	1	1	1	0	**12**
Rodrigues et al. [50]	1	1	1	2	1	1	1	0	0	0	1	1	1	1	0	**12**
Shi et al. [57]	1	1	1	1	1	1	1	0	0	0	1	1	1	1	0	**12**
Silva et al. [19]	1	1	1	1	1	1	1	0	0	0	1	1	1	1	0	**11**
Silva et al. [47]	1	1	1	1	1	1	1	0	0	0	1	1	1	0	0	**10**
Steinberg et al. [33]	1	1	1	1	1	1	1	0	0	0	1	1	1	0	0	**9**
Van Cant et al. [35]	1	1	1	1	1	1	1	0	0	0	1	1	1	0	0	**10**
Valle et al. [52]	1	1	1	1	1	1	1	1	0	0	0	1	1	1	0	**11**
Willson et al. [6]	1	1	1	2	1	1	1	0	0	0	1	1	1	0	0	**11**

*Note:* (1) clear objective or hypothesis; (2) outcomes clearly described; (3) patient characteristics clearly described; (5) confounding variables described^∗^; (6) main results clearly reported; (7) estimates of random variability provided; (10) actual probability values reported; (11) participants invited to participate representative of the target population; (12) participants who agreed to participate representative of the target population; (15) blinding of the outcome assessor; (16) analysis conducted as planned; (18) appropriate statistical analyses; (20) valid and reliable outcome measures; (21) appropriate case–control matching; and (25) adjustments made for confounding variables^∗^. For items 1–3, 6, 7, 10–12, 15, 16, 18, 20, 21, and 25, the response options were 0 (*no*), 1 (*yes*), or U (*unable to determine*). For item 5, the response options were 0 (*no*), 1 (*partially*), or 2 (*yes*). Bold values indicate the total score of the Downs and Black scale.

^∗^It was considered as confounding factors: sex, functional score, pain intensity, and duration of symptoms.

### 3.4. Meta‐Analysis Results

#### 3.4.1. Rearfoot Eversion

**FIGURE 2:**
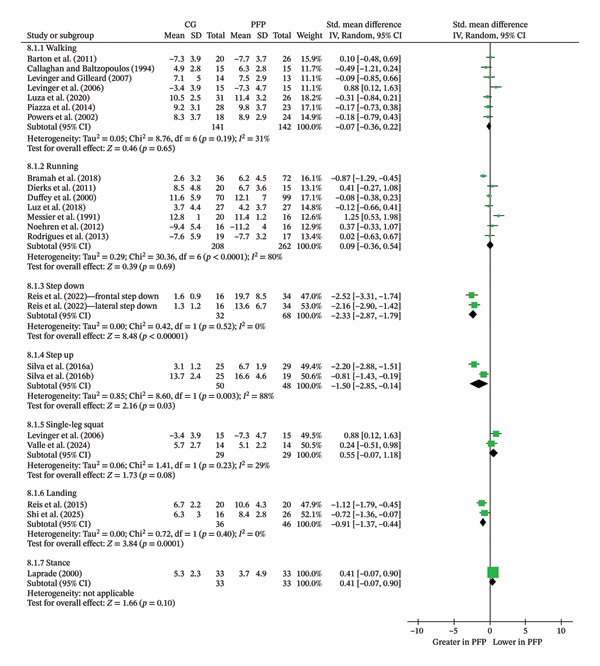
Rearfoot eversion angle, indicating whether it is greater or lower in individuals with PFP compared to those without PFP (CG).

No significant between‐group differences were observed during walking (SMD = −0.07; 95% CI: −0.36 to 0.22; *p* = 0.65; *I* ^2^ = 31%), running (SMD = 0.09; 95% CI: −0.36 to 0.54; *p* = 0.65; *I* ^2^ = 80%), or the single‐leg squat (SMD = 0.55; 95% CI: −0.07 to 1.18; *p* = 0.08; *I* ^2^ = 29%). Greater rearfoot eversion was observed in the PFP group during landing (SMD = −0.91; 95% CI: −1.37 to −0.44; *p* = 0.001; *I* ^2^ = 0%), step‐up (SMD = −1.50; 95% CI: −2.85 to −0.14; *p* = 0.03; *I* ^2^ = 88%), and step‐down (SMD = −2.33; 95% CI: −2.87 to −1.79; *p* < 0.001; *I* ^2^ = 0%). However, these findings should be interpreted with caution because only two studies contributed to these analyses, and the step‐up and step‐down outcomes were derived from the same participant sample in one study, limiting the independence of the evidence (Figure 2).

A leave‐one‐out sensitivity analysis was performed for the walking task. Excluding one study [49] reduced heterogeneity from 31% to 0% while the pooled effect remained nonsignificant (SMD = −0.18; 95% CI: −0.43 to 0.07; *p* = 0.15). For the running task, sequential removal of individual studies did not materially affect either the pooled effect estimate or the level of heterogeneity, suggesting that the observed variability could not be attributed to a single study. According to the predefined criteria, the level of evidence was classified as conflicting for walking, running, and the single‐leg squat and very limited for the landing, step‐up, and step‐down tasks.

#### 3.4.2. Ankle Dorsiflexion ROM Assessed by Kinematics

**FIGURE 3:**
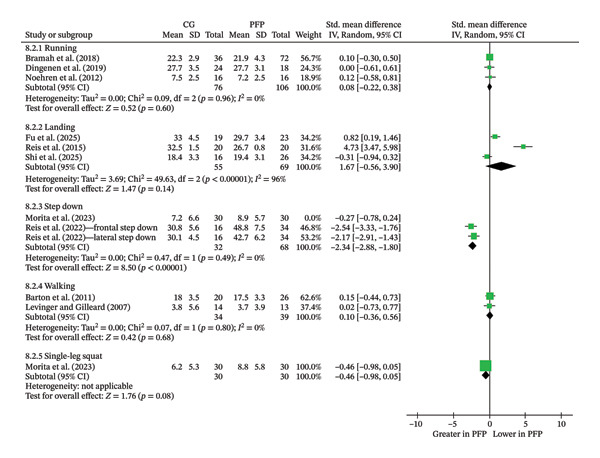
Ankle dorsiflexion ROM assessed by kinematics, indicating whether it is greater or lower in individuals with PFP compared to those without PFP (CG).

No significant between‐group differences were observed for walking (SMD = 0.10; 95% CI: −0.36 to 0.56; *p* = 0.68; *I* ^2^ = 0%), running (SMD = −0.08; 95% CI: −0.22 to 0.38; *p* = 0.60; *I* ^2^ = 0%), or landing (SMD = 1.67; 95% CI: −0.56 to 3.90; *p* = 0.14; *I* ^2^ = 96%). Greater ankle dorsiflexion was observed in the PFP group during the step‐down task (SMD = −1.64; 95% CI: −3.15 to −0.12; *p* = 0.03; *I* ^2^ = 93%). However, these findings should be interpreted with caution because they were based on only two studies, and the step‐down data were derived from the same participant sample reported in both studies, limiting the independence of the evidence (Figure 3).

A leave‐one‐out sensitivity analysis was conducted for the step‐down task. Excluding one study [60] substantially reduced heterogeneity (*I* ^2^ = 0%) while the pooled effect remained significant (SMD = −2.34; 95% CI: −2.88 to −1.80; *p* < 0.001). Nevertheless, the remaining evidence was derived from the same participant sample, thereby limiting the robustness of this finding despite the reduction in heterogeneity. For the landing task, the leave‐one‐out sensitivity analysis did not materially affect either the pooled effect estimate or the level of heterogeneity (Figure 3). According to the predefined criteria, the level of evidence was classified as conflicting for the walking, running, and landing tasks, whereas the evidence for the step‐down task was considered very limited because it was based on a small number of non‐independent studies.

#### 3.4.3. Ankle Dorsiflexion ROM Using Clinical Tests

**FIGURE 4:**
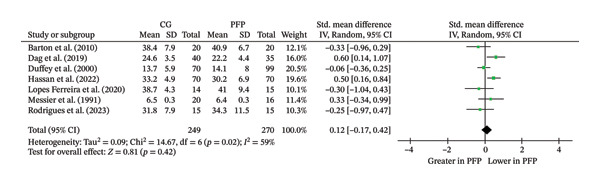
Ankle dorsiflexion ROM assessed by clinical tests, indicating whether it is greater or lower in individuals with PFP compared to those without PFP (CG).

No significant between‐group differences were observed (SMD = 0.12; 95% CI: −0.17 to 0.42; *p* = 0.42), although moderate heterogeneity was detected (*I* ^2^ = 59%) (Figure 4). Restricting the analysis to studies using passive measures did not alter the findings (SMD = 0.08; 95% CI: −0.26 to 0.42; *p* = 0.65), and heterogeneity remained moderate (*I* ^2^ = 54%). The leave‐one‐out sensitivity analysis also failed to identify any individual study responsible for the observed heterogeneity, indicating that the between‐study variability likely reflects methodological or clinical differences across studies rather than the influence of a single investigation. According to the predefined criteria, the level of evidence was classified as conflicting.

#### 3.4.4. Navicular Drop

**FIGURE 5:**
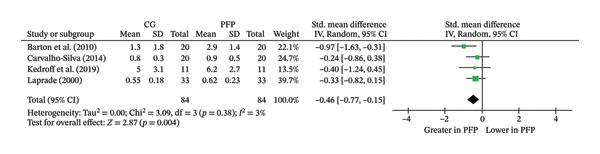
Navicular drop, indicating whether it is greater or lower in individuals with PFP compared to those without PFP (CG).

Greater navicular drop was observed in the PFP group (SMD = −0.46; 95% CI: −0.77 to −0.15; *p* = 0.004). This pooled estimate, derived from four studies involving 168 participants (PFP, *n* = 84; CG, *n* = 84), demonstrated low between‐study heterogeneity (*I* ^2^ = 3%), supporting the consistency of the observed association (Figure 5). The level of evidence was considered strong based on established criteria.

#### 3.4.5. Foot Posture Index

**FIGURE 6:**
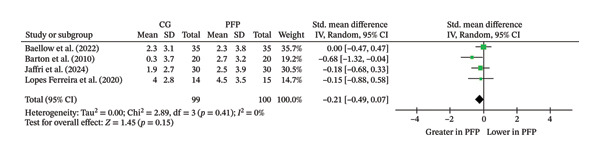
Foot posture index, indicating whether it is greater or lower in individuals with PFP compared to those without PFP (CG).

No significant between‐group differences were observed in the foot posture index (SMD = −0.21; 95% CI: −0.49 to 0.07; *p* = 0.15). Heterogeneity was negligible (*I* ^2^ = 0%), indicating consistent findings across the included studies (Figure 6). According to the predefined criteria, the level of evidence was classified as conflicting.

#### 3.4.6. Plantar Pressure

**FIGURE 7:**
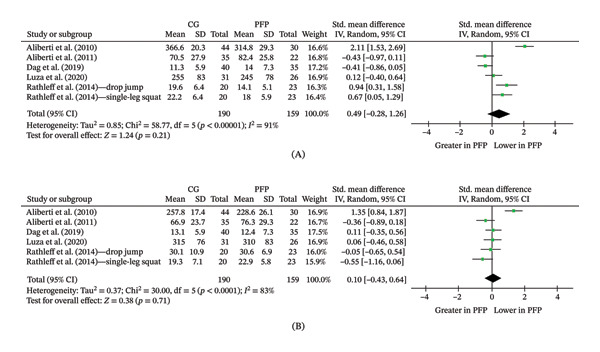
Plantar pressure in the lateral forefoot (A) and medial forefoot (B), indicating whether it is greater or lower in individuals with PFP compared to those without PFP (CG).

**FIGURE 8:**
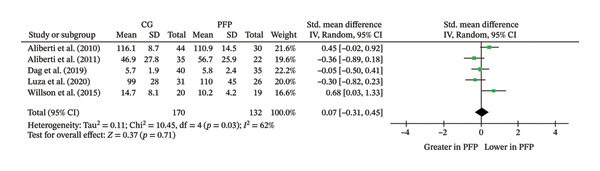
Plantar pressure in the midfoot, indicating whether it is greater or lower in individuals with PFP compared to those without PFP (CG).

**FIGURE 9:**
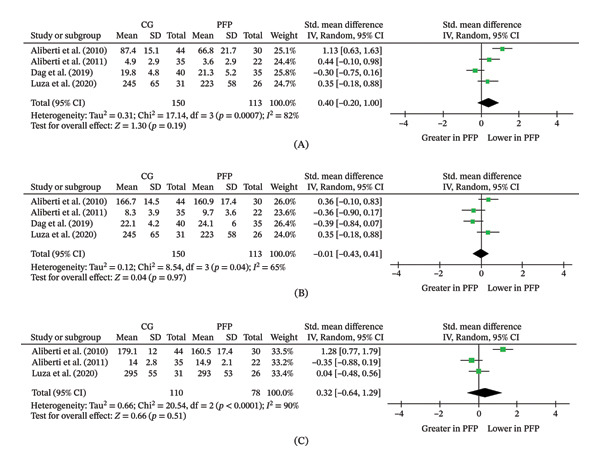
Plantar pressure in the lateral rearfoot (A), medial rearfoot (B), and central rearfoot (C), indicating whether it is greater or lower in individuals with PFP compared to those without PFP (CG).

No significant between‐group differences in plantar pressure were observed for the lateral forefoot (SMD = 0.49; 95% CI: −0.28 to 1.26; *p* = 0.21; *I* ^2^ = 91%) or medial forefoot (SMD = 0.10; 95% CI: −0.43 to 0.64; *p* = 0.71; *I* ^2^ = 83%) (Figure 7A–B), the midfoot (SMD = 0.07; 95% CI: −0.31 to 0.45; *p* = 0.71; *I* ^2^ = 62%) (Figure 8), or the lateral rearfoot (SMD = 0.40; 95% CI: −0.20 to 1.00; *p* = 0.19; *I* ^2^ = 82%), medial rearfoot (SMD = −0.01; 95% CI: −0.43 to 0.41; *p* = 0.97; *I* ^2^ = 65%), and central rearfoot (SMD = 0.32; 95% CI: −0.64 to 1.29; *p* = 0.51; *I* ^2^ = 90%) (Figure 9A–C). However, substantial between‐study heterogeneity was observed across all plantar pressure analyses (*I* ^2^ = 62%–91%), suggesting important methodological and/or clinical variability among the included studies. Accordingly, these findings should be interpreted with caution. The leave‐one‐out sensitivity analysis did not identify any single study responsible for the observed heterogeneity in most analyses. The only exception was the medial forefoot, where exclusion of one study [20] reduced heterogeneity from 83% to 2% without altering the overall conclusion, as the pooled effect remained nonsignificant (SMD = −0.12; 95% CI: −0.36 to 0.12; *p* = 0.32). According to the predefined criteria, the level of evidence was classified as conflicting.

#### 3.4.7. Muscle Strength

We were unable to perform a meta‐analysis of muscle strength because no muscle group was assessed by at least two studies using comparable methods. One study evaluated inversion and dorsiflexion strength [56], whereas two studies assessed plantarflexor strength using different outcome measures: One reported the number of repetitions completed during a heel‐rise test [35], while the other measured maximal plantarflexor strength with a hand‐held dynamometer [72]. In addition, the authors of the latter study did not provide the data required for quantitative synthesis [72].

## 4. Discussion

The findings of this systematic review and meta‐analysis suggest that individuals with PFP exhibit some differences in distal joint biomechanics compared with asymptomatic controls. Strong evidence supports greater navicular drop in individuals with PFP; however, this conclusion is based on only four studies, despite the low between‐study heterogeneity. Greater rearfoot eversion during landing and step‐down tasks, as well as greater ankle dorsiflexion during the step‐down task, were also observed in individuals with PFP. However, these findings should be interpreted with caution because they were derived from a limited number of studies, some of which included the same participant sample, and substantial between‐study heterogeneity was observed in some analyses. Accordingly, these outcomes were classified as providing very limited evidence. In contrast, no between‐group differences were observed for ankle dorsiflexion ROM measured in clinical tests, foot posture index scores, or plantar pressure distribution. Nevertheless, the findings for plantar pressure should also be interpreted cautiously because of the considerable between‐study heterogeneity. Finally, a quantitative synthesis of muscle strength could not be performed because no muscle group was evaluated by at least two studies using comparable assessment methods.

We found strong evidence supporting greater navicular drop in individuals with PFP; however, this conclusion is based on only four studies. Although the evidence synthesized in this review was derived predominantly from case–control studies, a prospective cohort study has suggested that greater navicular drop may be a risk factor for the development of PFP, providing preliminary support for this association [73]. This finding is biomechanically plausible, as greater navicular drop reflects increased foot pronation, which has been proposed as a mechanism that may increase patellofemoral joint stress through alterations in lower‐limb kinematics and, consequently, contribute to the development of PFP [3].

The greater navicular drop observed in individuals with PFP may reflect increased foot pronation and reduced stability of the medial longitudinal arch. Biomechanically, excessive foot pronation has been proposed to alter the alignment of the lower‐limb kinetic chain, increasing rearfoot eversion, tibial internal rotation, and hip adduction [3, 74]. These coupled movements may facilitate the development of dynamic knee valgus, which has been associated with greater pain severity in women with PFP [75]. Consistent with this proposed mechanism, our meta‐analysis also identified greater rearfoot eversion during landing and step‐down tasks, as well as greater ankle dorsiflexion during the step‐down task, in individuals with PFP. However, these findings were supported by very limited evidence because they were derived from a small number of studies, some of which included the same participant sample, and substantial heterogeneity was observed in some analyses. Interestingly, a previous meta‐analysis also reported limited evidence of greater rearfoot eversion during gait in women with PFP [22], providing additional support for the potential role of altered foot biomechanics in this population. Which may contribute to pain severity in female participants with PFP.

Ankle dorsiflexion ROM measured using clinical tests did not differ between individuals with and without PFP. Similarly, no between‐group differences were observed during walking, running, or single‐leg squat tasks, whereas greater ankle dorsiflexion was identified only during the step‐down task, although this finding was supported by very limited evidence. Previous evidence has reported a moderate association between reduced ankle dorsiflexion and increased dynamic knee valgus [76], suggesting that limited dorsiflexion may contribute to compensatory movement patterns. However, our findings do not consistently support this mechanism. The discrepancies across studies are likely explained by differences in assessment methods, task demands, participant characteristics, and the multifactorial nature of PFP, in which biomechanical alterations may not be consistently present across all individuals [77].

Regarding plantar pressure, the meta‐analysis did not identify consistent differences in load distribution across the forefoot, midfoot, or rearfoot between individuals with and without PFP. However, these findings should be interpreted with caution because substantial between‐study heterogeneity was observed across all analyses (I^2^ = 62%–91%) and persisted after sensitivity analyses for most outcomes, suggesting that the variability could not be attributed to any single study. Although it has been hypothesized that individuals with PFP adopt compensatory weight‐bearing strategies to minimize pain [20], these adaptations may vary considerably across individuals and functional tasks, limiting the consistency of plantar pressure findings. This variability may reflect differences in movement strategies [78], task demands [79], and methodological differences in plantar pressure assessment across studies. Therefore, although no significant between‐group differences were identified, the considerable heterogeneity reduces confidence in these pooled estimates, indicating that additional studies using standardized plantar pressure assessment protocols are needed.

An important aspect was the inability to perform a meta‐analysis for muscle strength outcomes. This is noteworthy, given that muscle strength is one of the most frequently addressed and studied clinical outcomes in PFP, both for hip muscles [11] and for knee muscles [14], but does not appear to be measured systematically in ankle and foot muscles. Therefore, we suggest that strength assessments of these muscles be included to better understand potential mechanisms that may explain the findings of the present meta‐analysis.

The main methodological limitations identified were the lack of assessor blinding and the absence of adjustment for potential confounding factors. Although the impact of assessor blinding in observational studies remains uncertain, evidence from randomized controlled trials suggests that the absence of blinding may overestimate intervention effects by approximately 13%, particularly for evaluator‐dependent outcomes [80]. More importantly, none of the included studies adjusted for potential confounders, such as pain intensity, symptom duration, physical activity level, or functional status. Previous research has shown that the number of kinematic alterations is strongly associated with pain intensity and AKPS scores [81], indicating that these variables may substantially influence between‐group comparisons. Furthermore, several studies did not consistently report key clinical characteristics, including pain intensity, functional status, and symptom duration (Table 2), precluding subgroup or meta‐regression analyses to investigate their potential influence on the pooled estimates. The inconsistent reporting of these variables, together with the lack of adjustment for confounding factors, likely contributed to the substantial between‐study heterogeneity observed in several meta‐analyses and should be considered when interpreting the present findings. Therefore, future studies should consistently report these clinical characteristics and account for potential confounding factors to improve the validity and comparability of biomechanical outcomes in individuals with PFP.

In our meta‐analysis, some methodological limitations should be considered when interpreting the present findings. First, for studies in which numerical data were not reported and could not be obtained from the authors, values were extracted from published figures using ImageJ software. Although this approach is commonly used in systematic reviews, it may have introduced measurement error. Second, when studies reported outcomes from multiple phases of the same movement or different subgroups within the same sample, these values were averaged before quantitative synthesis to avoid multiple non‐independent comparisons. While this approach minimized unit‐of‐analysis issues, it may have reduced within‐study variability and masked phase‐specific or subgroup‐specific differences.

From a clinical perspective, these findings reinforce the importance of a comprehensive assessment of individuals with PFP, including the evaluation of distal joint biomechanics as part of the entire lower‐limb kinetic chain. In particular, greater navicular drop may represent a clinically relevant characteristic, as it was the only outcome supported by strong evidence, although this conclusion was based on only four studies. Conversely, alterations in rearfoot eversion and ankle dorsiflexion during functional tasks should be interpreted with caution because the available evidence is very limited. Furthermore, causality cannot be established due to the predominantly case–control design of the included studies. Future prospective studies and randomized clinical trials are needed to clarify the clinical relevance of these biomechanical characteristics and to identify which patients are most likely to benefit from distal joint–targeted interventions.

## 5. Conclusion

This systematic review and meta‐analysis suggest that altered distal joint biomechanics are associated with PFP, particularly greater navicular drop, for which strong evidence was identified despite the limited number of available studies. Evidence for greater rearfoot eversion and ankle dorsiflexion during functional tasks was considerably weaker because of methodological limitations, substantial heterogeneity, and the small number of independent studies. No consistent differences were observed in ankle dorsiflexion ROM measured clinically, foot posture index, or plantar pressure distribution. These findings highlight the need for well‐designed prospective studies using standardized biomechanical assessments to determine whether distal joint impairments represent modifiable risk factors or secondary adaptations in individuals with PFP.

## Funding

No funding was received for this manuscript.

## Conflicts of Interest

The authors declare no conflicts of interest.

## Supporting Information

Additional supporting information can be found online in the Supporting Information section.

## Supporting information


**Supporting Information** PRISMA 2020 checklist.

## Data Availability

The data that support the findings of this study are available from the corresponding author upon reasonable request.
